# Mesenchymal stem cell therapy for therapy refractory complex Crohn’s perianal fistulas: a case series

**DOI:** 10.1186/s13287-024-03779-0

**Published:** 2024-06-09

**Authors:** A. J. M. Pronk, K. J. Beek, M.E. Wildenberg, W. A. Bemelman, J. Stoker, C. J. Buskens

**Affiliations:** 1 Department of Surgery, Amsterdam UMC, location VUMC, De Boelelaan 1117, 1081HV, Amsterdam, The Netherlands; 2 Department of Radiology and Nuclear Medicine, Amsterdam UMC, University of Amsterdam, Amsterdam, The Netherlands; 3 Amsterdam UMC, Tytgat Institute for Liver and Intestinal Research, University of Amsterdam, Amsterdam, The Netherlands; 4 Amsterdam Gastroenterology Endocrinology Metabolism, Amsterdam, the Netherlands

**Keywords:** Perianal fistulas, Crohn’s disease, Mesenchymal stem cells

## Abstract

**Background:**

Mesenchymal stem cell treatment (MST) has emerged as a new therapeutic strategy for Crohn’s perianal fistulas. It has been demonstrated that a fibrotic tract on MRI with a MAGNIFI-CD score ≤ 6 is the best predictor for long-term clinical closure. Therefore, the aim of the current study was to analyse the effectiveness of MST for complex Crohn’s perianal fistulas based on MRI.

**Methods:**

Consecutive patients with complex Crohn’s perianal fistulas, previously failing both anti-TNF treatment and surgical closure, who had surgical closure of the internal opening with MST were included. The primary endpoint was radiological remission of the fistula(s) defined as a MAGNIFI-CD ≤ 6 on MRI, read by an experienced radiologist.

**Results:**

Between December 2019 and March 2023, 30 patients (15 males) with 48 fistula tracts were included with a median follow-up of 16.5 months. Radiological remission was achieved in thirteen patients (43.3%) after a median follow-up of 5.0 months (IQR 3.0–6.0). The median MAGNIFI-CD at baseline was 15.0 (IQR 7.0–20.0) which significantly decreased to 8.0 (IQR 3.0–15.0) after treatment (*p* = 0.001). Clinical closure was achieved in 21 patients (70.0%). Three patients (14.3%) developed a recurrence during long-term FU, all with clinically closed fistula(s), but no radiological remission. The median PDAI decreased significantly from 10.5 (IQR 7.0–14.0) to 4.0 (IQR 0.0-7.3) (*p* = 0.001).

**Conclusion:**

MST is a promising treatment strategy for therapy refractory Crohn’s perianal fistulas, resulting in > 40% radiological remission, clinical closure in 70% and a significant improvement in quality of life. No recurrences were seen in patients with radiological remission.

## Introduction

Perianal fistulas are a common complication of Crohn’s disease (CD). Almost 30% of the patients with CD will develop one or more perianal fistulas within the first two decades after diagnosis, with the majority of these fistulas being complex [1]. Frequently reported complaints due to perianal fistulas are pain and drainage of pus, stool or blood, severely impacting quality of life [2]. In addition, 20% of the patients experience faecal incontinence due to damage of the anal sphincter [3]. The treatment for Crohn’s perianal fistulas consists of a combination of medical and surgical therapies [4]. However, treatment options are limited and characterised by high recurrence rates after both medical and surgical approaches. Thus, a more effective, alternative treatment is necessary.

Mesenchymal stem cell treatment (MST) as a reinforcement of surgical closure of the internal opening has emerged as a new therapeutic strategy. Stem cells are capable of affecting the inflammatory processes and reconstructing tissue [5, 6]. The randomized controlled trial (RCT) of Panés et al. [7] compared MST using Darvadstrocel and placebo, showing a significant higher remission rate at 24 weeks follow-up in the MST group vs. the placebo group (50% vs. 34%). This finding was maintained at 52 weeks [8]. However, when analysing long term follow-up, a recurrence rate of > 50% was seen, which was comparable in both MST and placebo group [9]. The high recurrence rate was probably related to the primary outcome parameter: combined clinical remission defined as clinical closure of external opening(s) and the absence of a collection larger than two centimetres on Magnetic Resonance Imaging (MRI). It has been demonstrated that only a completely fibrotic fistula tract on MRI with a MAGNIFI-CD ≤ 6 is the best predictor for long-term clinical closure [10, 11]. As long-term outcomes are most relevant, a different primary endpoint with radiological remission and a MAGNIFI-CD ≤ 6 on MRI should be strived for. Based on this, the aim of this study was to analyse the effectiveness of surgical closure and MST for complex perianal fistulas in patients with CD based on radiological imaging.

## Methods

### Study design

In this retrospective case series, consecutive patients with complex Crohn’s perianal fistulas, previously failing both anti-TNF treatment and surgical closure, that underwent surgical closure of the internal opening combined with MST between December 2019 and March 2023 were included. There were no limitations or restrictions for inclusion. The Medical Ethical Committee at the Amsterdam UMC confirmed no need for ethical approval. All participants signed informed consent.

### Patient demographics and outcome variables

Patient demographics, previous fistula therapy including hyperbaric oxygen therapy, medication and surgeries, and details of the MST and follow-up were collected from medical notes. Patient demographics included sex, age, smoking history, duration of CD, location of CD according to the Montreal classification, and fistula characteristics. The primary outcome parameter was radiological fistula remission, defined as a MAGNIFI-CD score ≤ 6 on MRI. Previous research showed that over 90% of the patients scored with a MAGNIFI-CD ≤ 6 have long-term clinical closure [11]. MRI’s were read by an experienced abdominal radiologist. Secondary endpoints were the change in MAGNIFI-CD scores pre- and postoperatively, clinical fistula closure, defined as closure of the external opening(s) and no production upon palpation, recurrence rate, quality of life based on the perianal disease activity index (PDAI) [12], serious adverse events (SAE) and reinterventions.

### Surgical intervention

To improve outcomes, all fistulas were pre-treated with seton drainage for at least six weeks and medical therapy was optimised if possible (preferably anti-TNF with through level > 10). The technical aspects of the MST procedure has been previously described [7]. First, the fistulas tract(s) were vigorously curetted, after which the seton was removed. Then, the internal and external opening were de-epithelised, and the internal opening was closed with absorbable vicryl 2.0 sutures. Closure of the internal opening was confirmed by injection of 10mL NaCl. Next, 24 mL of darvadstrocel, a cell suspension containing 120 × 10^6^ cells (expanded adipose derived stem cells; Cx601) as a single intralesional dose was injected. The first half of the dose was injected around the sutured internal opening(s) through the anal canal, and the other half was injected into the tract wall along the fistula through the external opening(s), resulting in several micro-blebs forming inside the tract wall.

### MRI protocol and assessment

MRI scans were acquired at 1.5 Tesla (Siemens Avanto Fit) or 3.0 Tesla (Philips Ingenia) scanners. Pelvic imaging protocol consisted of sagittal and coronal T2-weighted sequence and axial T2-weigthed sequence and axial T1-weigthed sequence with fat-suppression after intravenously contrast administration (0.1 mmol/kg). Coronal and axial images were angulated parallel and perpendicular to the anal canal, respectively. Before scanning patients received intravenous antiperistaltic medication (Scopolamine butylbromide, Buscopan; AS KALCEKS, Riga, Letland). Pre-operative MRI scans were collected to assess baseline MRI characteristics. The most recent MRI before MST was used. Most recent acquired post-operative MRI’s with at least 3 months interval after MST were collected. Previously, it was demonstrated that performing a MRI three months after surgery was relevant to assess fistula healing [13]. One expert abdominal radiologist (JS) with more than 25 years’ experience with perianal CD fistula MRIs scored the individual items of the MAGNIFI-CD index, where after the total scores were calculated [14]. Scoring items and their weights are shown in Table 1.

**Table 1:** MAGNIFI-CD Index [14]

Item	Characteristics	Weight
No. of fistula tracts	0 = None 1 = Single, unbranched 2 = Complex	x3
Hyperintensity of primary tract on post-contrast T1-weighted images	0 = Absent/mild 1 = Pronounced	x2
Dominant feature	0 = Predominantly fibrous 1 = Predominantly granulation tissue 2 = Predominantly fluid/pus	x2
Fistula length	0 = < 2.5 cm 1 = 2.5–5 cm 2 = > 5 cm	x2
Extension	0 = Absent 1 = Horseshoe 2 = Infralevatoric/supralevatoric	x2
Inflammatory mass	0 = Absent 1 = Focal 2 = Diffuse 3 = Collections, small 4 = Collections, medium 5 = Collections, large	x1

### Statistical analysis

Patients characteristics were summarized using descriptive statistics; median and interquartile range (IQR) were used to present non-parametric data, and frequencies and percentages were used to present categorical data. The primary outcome was summarized using descriptive statistics. The Wilcoxon signed rank test was used to analyse secondary outcomes. Patient and fistula characteristics associated with radiological remission were analysed by means of logistic regression analyses.

## Results

### Baseline characteristics

A total of 30 patients with 48 fistula tracts were included (Table 2). The median age at surgery was 34.5 years (IQR 25.8–43.3) and half of the patients (50.0%) were male. The median disease duration of CD was 8.5 years (IQR 6.8–13.0), and the most common disease location was ileocolic (40.0%). Six patients (20.0%) were smoking, and 29 patients (96.7%) were using medication of which the majority used anti-TNF treatment (83.3%). One third of the patients (33.3%) had a defunctioning ostomy and six patients (20.0%) underwent hyperbaric oxygen therapy in the past. The median fistula duration was 4.0 years (IQR 2.0-8.3) and the median number of perianal procedures in the study population was 6.0 (IQR 3.0-7.3). Median duration of pre-treatment seton drainage was 10.0 months (IQR 7.0-25.5). Most patients, (60.0%) had one external opening at time of surgery, followed by two (23.3%), three (10.0%), and four (6.7%) external openings.

**Table 2:** Baseline characteristics

	*n* = 30
**Male sex, ** *n* ** (%)**	15 (50.0%)
**Age, years** ^**a**^	34.5 (25.8–43.3)
**Smoking at moment of surgery, n (%)**	6 (20.0%)
**Duration Crohn’s disease, years** ^**a**^	8.5 (6.8–13.0)
**Location of Crohn’s disease, n (%)**	
L0	1 (3.3%)
L1 Ileum	7 (23.3%)
L2 Colon	7 (23.3%)
L3 Ileum and colon	12 (40%)
L1-L4	2 (6.7%)
**Use of medication, n (%)**	
Anti-TNF treatment	21 (70.0%)
Immunomodulator	4 (13.3%)
Combination of anti-TNF treatment and immunomodulator	4 (13.3%)
None	1 (3.3%)
**Previous abdominal surgery, n (%)**	19 (63.3%)
**Duration of perianal fistulas, years** ^**a**^	4.0 (2.0-8.3)
**Number of perianal operations in the past** ^**a**^	6.0 (3.0-7.3)
**HBO before MST, n (%)**	6 (20.0%)
**Seton drainage time, months** ^**a**^	10.0 (7.0-25.5)
**Ostomy at time of surgery, n (%)**	10 (33.3%)
**Internal openings, n (%)**	
1	26 (86.7%)
2	3 (10.0%)
3	1 (3.3%)
**External openings, n (%)**	
1	18 (60.0%)
2	7 (23.3%)
3	3 (10.0%)
4	2 (6.7%)

a Values are presented median (interquartile range)

### Fistula Closure

**Fig. 1:**
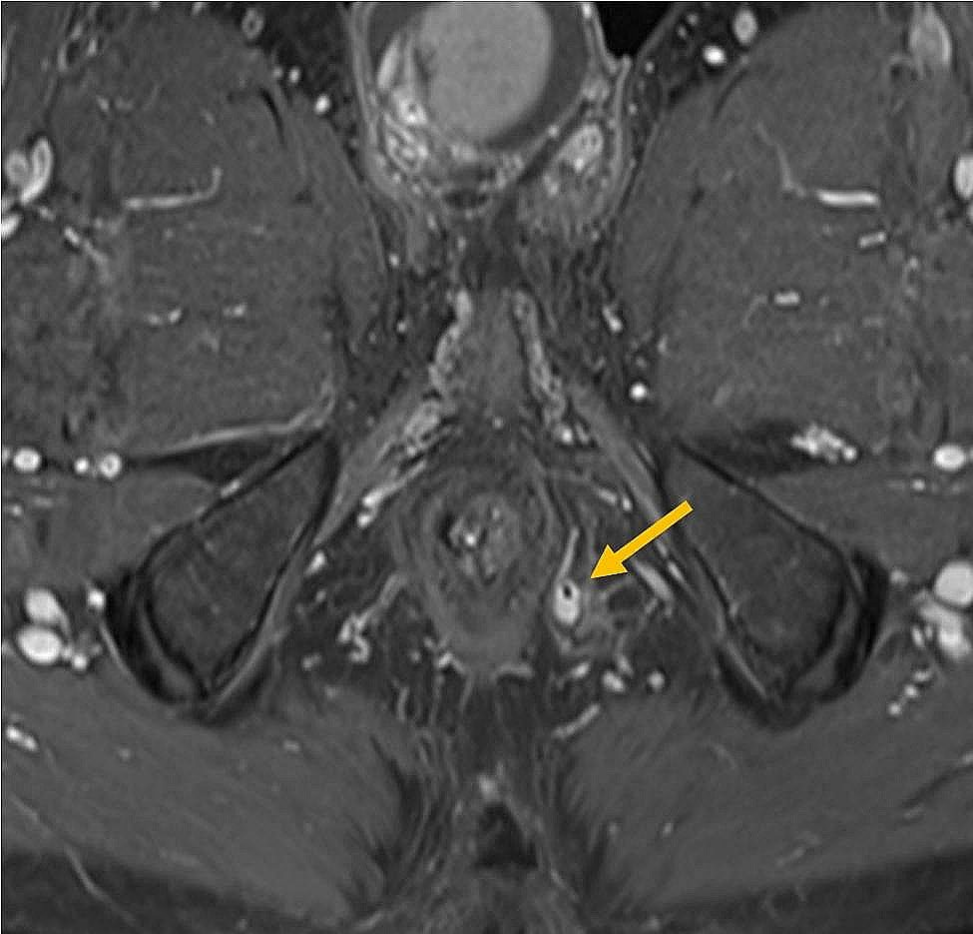
a. Pre-treatment MRI: An axial T1-weighted sequence with fat suppression after contrast administration with a total MAGNIFI-CD score of 18 based on a complex fistula longer than 5 cm with intralevatoric extension, pronounced T1 hyperintensity and for more than 50% consisting of granulation tissue. The active fistula is indicated with an orange arrow

Postoperative MRI demonstrated radiological remission in thirteen patients (43.3%), after a median time of 5.0 months (IQR 3.0–6.0). The median MAGNIFI-CD at baseline was 15.0 (IQR 7.0–20.0) and decreased significantly to a median of 8.0 (IQR 3.0–15.0; *p* = < 0.001) (Fig. 1a and b). Clinical closure of the fistula(s) was seen in 21 patients (70.0%) after a median time of 1.0 month (IQR 1.0-2.5). During follow-up (median 16.5 months, IQR 11.0-22.3), three out of 21 clinically closed patients (14.3%) developed a clinical recurrence. These three patients did not achieve radiological remission. No patient or fistula characteristics, including defunctioning ostomy at time of surgery, number of perianal operations in the past, and number of internal- and external fistula openings, could be associated to radiological remission.

**Fig. 2:**
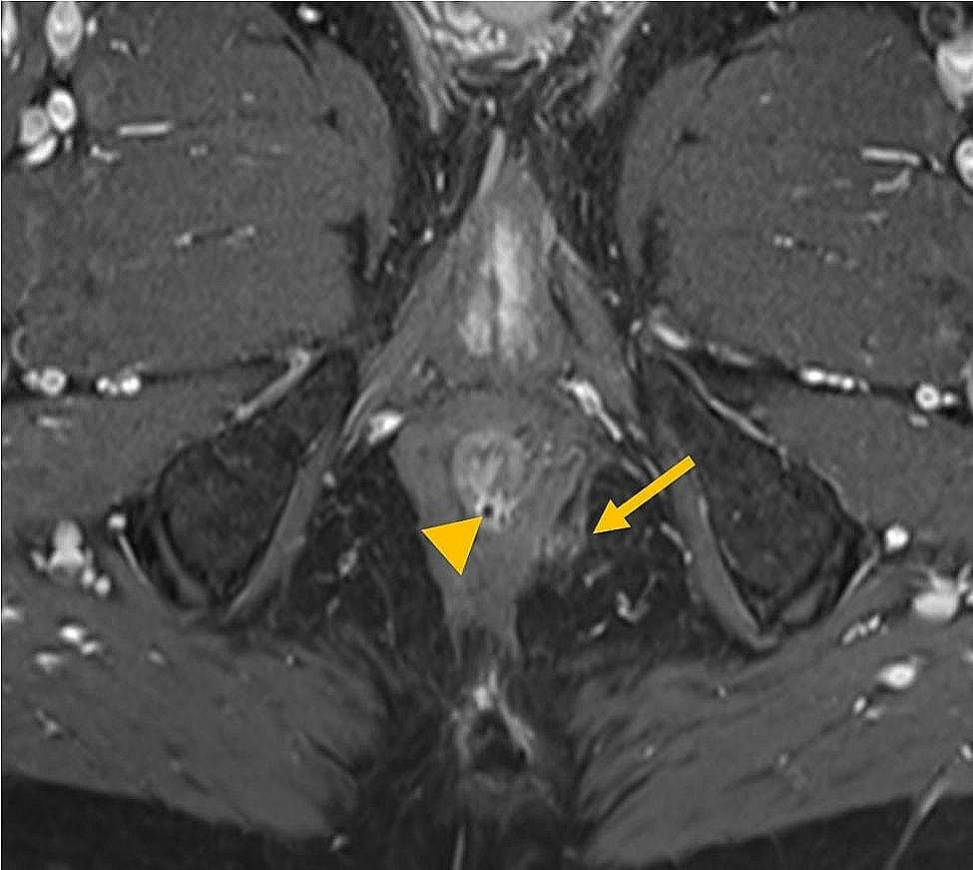
b. Post-treatment MRI: An axial T1-weighted sequence with fat suppression after contrast administration with a total MAGNIFI-CD score of 5 based on a single fistula tract with a fistula length between 2.5 and 5 cm, without extension, mild T1 hyperintensity and more than 50% fibrotic tissue. Artefact in the posterior intersphincteric space (arrow head) caused by the MSC treatment

### Quality of life

The median PDAI was 10.5 (IQR 7.0–14.0) at baseline. This index score decreased significantly to a median of 4.0 (IQR 0.0-7.3) after a median follow-up of 9.0 months (IQR 5.0–11.0; *p* = < 0.001) (Table 3). All separate elements of the PDAI decreased significantly as well (*p* = < 0.001 for all), with the largest median decrease in discharge. The median PDAI in patients with radiological remission decreased from 9.0 (IQR 7.0-11.5) at baseline to 0.0 (IQR 0.0–3.0) and from 11.0 (IQR 9.0–15) to 6.0 (IQR 2.5-9.0) in patients without radiological remission.

**Table 3:** PDAI

	Baseline	Follow-up	*P*
**Discharge**	2.5 (1.0–3.0)	0.0 (0.0–1.0)	*p* = < 0.001
**Pain and restriction of activities**	2.0 (2.0–3.0)	1.0 (0.0–2.0)	*p* = < 0.001
**Restriction of sexual activity**	2.0 (2.0–2.0)	1.0 (0.0–2.0)	*p* = < 0.001
**Type of perianal disease**	2.0 (2.0–2.0)	0.0 (0.0–2.0)	*p* = < 0.001
**Degree of induration**	1.5 (1.0–2.0)	0.0 (0.0–0.0)	*p* = < 0.001
**Total PDAI**	10.5 (7.0–14.0)	4.0 (0.0-7.3)	*p* = < 0.001
**Total PDAI in patients with radiological remission**	9.0 (7.0-11.5)	0.0 (0.0–3.0)	*P* = 0.001
**Total PDAI in patients without radiological remission**	11.0 (9.0–15.0)	6.0 (2.5-9.0)	*p* = < 0.001

### Serious adverse events and reinterventions

Postoperatively, one serious adverse event occurred. In this patient a necrotic wound developed after MST injection in a relatively short anterior fistula tract, probably due to pressure necrosis, resulting in a rectovaginal fistula, necessitating temporary defunctioning. After a sphincter repair the fistula was clinically closed with radiological remission, after which the ostomy was reversed without complications. Another six patients underwent a fistula related reintervention due to a postoperative abscess or an ongoing/recurrent fistula tract(s) after MST. All were treated with seton drainage and two patients were retreated with stem cells, resulting in clinical closure in both patients and radiological remission in one.

## Discussion

This case series shows the radiological outcomes after surgical closure of the internal opening combined with MST in 30 patients with Crohn’s perianal fistulas. In 43%, this procedure resulted in radiological remission after a median follow-up of 5.0 months. The median MAGNIFI-CD decreased significantly from 15.0 to 8.0 (*p* = < 0.001). Clinical closure was seen in 70% of the patients, with only 14% recurrence. The current closure and recurrence rates are slightly better than the previously presented results in the ADMIRE trial, where 50% combined clinical and radiological remission in the intervention group was seen, with > 50% recurrences within three years [7, 9]. The definition of radiological remission in the ADMIRE trial, the absence of a collection > 2 cm on MRI, was probably related to the disappointing long-term outcomes. Although we only have a median follow-up of 16.5 months in this case series, the relatively high rate of radiological remission is probably related to the low recurrence rate, as it has been previously demonstrated that no recurrences are seen in patients with radiological remission [10].

The results are even more remarkable considering the fact that this was a therapy-refractory patient group with a median CD duration of 8.5 years, median fistula duration of 4.0 years and a median of 6.0 previous surgical fistula interventions. Although it is difficult to speculate why the results are better in the current series, it does not seem to be related to a defunctioning ostomy, as outcomes were comparable between defunctioned and non-defunctioned patients. It can be hypothesized that the results reflect specialised care in a tertiary referral centre for Crohn’s fistulas, with all patients optimised in medical treatment, pre-treated with seton drainage, and extensive surgical experience with closure of the internal opening. Other single centre series confirm the possibility of good results after closure of the internal opening combined with MST. A French prospective cohort study investigating 27 patients treated with stem cell injections found a clinical closure rate of 52% and a combined clinical- radiological response of 35% [15]. The results are also in line with the results from the PISAII trial, where radiological healing was seen in 32% of the surgical closure group (advancement plasty or ligation of the intersphincteric fistula tract) with 68% clinical closure [10]. This further emphasizes the potential of inducing radiological remission in therapy-refractory patients with good long-term results after MST, as the current group all previously underwent surgical closure in the same centre.

The median decrease of 7.0 points in the MAGNIFI-CD score in the current series supports the clinical findings. A study by Beek et al. [16] demonstrated that a decrease of 2.0 points in the MAGNIFI-CD can be used as a cut-off for clinical response. Although this validation cohort did not include patients treated with MST, the median decrease in MAGNIFI-CD in this case-series could be considered as highly clinically relevant. Moreover, the absolute MAGNIFI-CD score in this patient group might even be an underestimation of the actual effect, as with this scoring technique only the worst feature is scored and one third of these patients had more than one fistula tract where a better result was not taken into account.

For patients with Crohn’s perianal fistulas, quality of life, rather than radiological outcomes, is the most important outcome parameter. The good clinical results of closure of the internal opening combined with MST were also reflected in the PDAI, which overall decreased from 10.5 to 4.0. Although the PDAI is not officially a patient reported outcome parameter, as it measures disease burden by patients and doctors combined, it is a validated score system that can be interpreted as quality of life [12]. It has been demonstrated that a PDAI ≤ 4 can be considered as inactive perianal disease [17]. In that light, the overall decrease in PDAI to 4.0 within nine months can be considered a good result and shows that even if surgical closure of the internal opening combined with MST is not capable of inducing radiological remission, patients benefit from this treatment. However, the more interesting finding is probably the median PDAI of 0.0 achieved in patients with radiological remission. This result is generally not seen after medical treatment alone, despite complete clinical closure. This highlights the importance of trying to achieve radiological remission in patients with Crohn’s perianal fistulas. The fact that no recurrences were seen in this group further emphasizes the clinical importance of aiming for radiological remission.

Obviously, as with all surgical interventions aiming for fistula closure, the MST procedure was not without (serious) adverse events. Six patients needed reinsertion of a seton due to a postoperative abscess or ongoing fistula symptoms (6/30 = 20%). This incidence is comparable to other series presenting results on advancement plasty or LIFT [10, 13]. One patient developed a necrotic wound and rectovaginal fistula due to pressure necrosis after MST. This complication was seen in a short, epithelialized, anterior fistula tract, which suggests that care should be taken when injecting the high volume of darvadstrocel (24 cc) in small fistulas.

A few limitations of the current study need to be considered. First, the small number of patients with only 30 patients included in three years. However, in the Netherlands, only patients with treatment refractory Crohn’s perianal fistulas failing both anti-TNF and surgical closure are eligible for treatment with stem cell injections, so this is a reflection of daily clinical practice. Second, data was collected retrospectively. Nonetheless, every patient treated with closure of the internal opening in combination with MST at the Amsterdam UMC receives the same close monitoring follow-up, including standard MRI after three to six months, thus the retrospective design did not lead to missing data. The strength of the study is the reading of MRIs by a specialised senior radiologist, which makes the primary endpoint independent of interpretation by the treating physician, which is a well-known confounder in generally used primary outcomes parameters like clinical closure and fistula drainage assessment, which were previously qualified as imprecise [18].

In conclusion, closure of the internal fistula opening in combination with MST is a promising treatment strategy for therapy refractory Crohn’s perianal fistulas, resulting in > 40% radiological remission and clinical closure in 70%. MST also resulted in improved quality of life with an overall significant decrease in PDAI to 4.0, reflecting inactive perianal disease. Moreover, in the group with radiological remission, a median PDAI of 0.0 was achieved, with no recurrences in long-term follow-up. This highlights the importance of striving for radiological remission in perianal fistula treatment. Research is needed to gain insight in which patients or fistula characteristics MST is most likely to induce radiological remission to further improve outcomes.

## Data Availability

The datasets used and/or analysed during the current study are available from the corresponding author on reasonable request.

## Abbreviations

CD: Crohn’s Disease
MST: Mesenchymal Stem Cell Treatment
MRI: Magnetic Resonance Imaging
PDAI: Perianal Disease Activity Index
SAE: Serious Adverse Events

## References

[1] Lahat A, Assulin Y, Beer-Gabel M, Chowers Y (2012). Endoscopic ultrasound for perianal Crohn’s disease: disease and fistula characteristics, and impact on therapy. J Crohns Colitis.

[2] Eglinton TW, Barclay ML, Gearry RB, Frizelle FA (2012). The spectrum of perianal Crohn’s disease in a population-based cohort. Dis Colon Rectum.

[3] Stewart DB (2017). Sr. Fecal incontinence among patients with Crohn’s Disease: does Awareness Change anything?. Dis Colon Rectum.

[4] Steinhart AH, Panaccione R, Targownik L, Bressler B, Khanna R, Marshall JK (2019). Clinical practice Guideline for the Medical Management of Perianal Fistulizing Crohn’s Disease: the Toronto Consensus. Inflamm Bowel Dis.

[5] Singer NG, Caplan AI (2011). Mesenchymal stem cells: mechanisms of inflammation. Annu Rev Pathol.

[6] Cao Y, Su Q, Zhang B, Shen F, Li S (2021). Efficacy of stem cells therapy for Crohn’s fistula: a meta-analysis and systematic review. Stem Cell Res Ther.

[7] Panés J, García-Olmo D, Van Assche G, Colombel JF, Reinisch W, Baumgart DC (2016). Expanded allogeneic adipose-derived mesenchymal stem cells (Cx601) for complex perianal fistulas in Crohn’s disease: a phase 3 randomised, double-blind controlled trial. Lancet.

[8] Panés J, García-Olmo D, Van Assche G, Colombel JF, Reinisch W, Baumgart DC (2018). Long-term efficacy and safety of Stem Cell Therapy (Cx601) for Complex Perianal Fistulas in patients with Crohn’s Disease. Gastroenterology.

[9] Panés J, Bouma G, Ferrante M, Kucharzik T, Nachury M, de la Portilla de Juan F (2022). INSPECT: a retrospective study to Evaluate Long-Term Effectiveness and Safety of Darvadstrocel in patients with Perianal Fistulizing Crohn’s disease treated in the ADMIRE-CD trial. Inflamm Bowel Dis.

[10] Meima-van Praag EM, van Rijn KL, Wasmann K, Snijder HJ, Stoker J, D’Haens GR (2022). Short-term anti-TNF therapy with surgical closure versus anti-TNF therapy in the treatment of perianal fistulas in Crohn’s disease (PISA-II): a patient preference randomised trial. Lancet Gastroenterol Hepatol.

[11] van Rijn KL, Meima-van Praag EM, Bossuyt PM, D’Haens GR, Gecse KB, Horsthuis K (2022). Fibrosis and MAGNIFI-CD activity index at Magnetic Resonance Imaging to Predict Treatment Outcome in Perianal Fistulizing Crohn’s Disease patients. J Crohns Colitis.

[12] Adegbola SO, Dibley L, Sahnan K, Wade T, Verjee A, Sawyer R (2021). Development and initial psychometric validation of a patient-reported outcome measure for Crohn’s perianal fistula: the Crohn’s Anal Fistula Quality of Life (CAF-QoL) scale. Gut.

[13] Meima-van Praag EM, van Rijn KL, Monraats MA, Buskens CJ, Stoker J (2021). Magnetic resonance imaging after ligation of the intersphincteric fistula tract for high perianal fistulas in Crohn’s disease: a retrospective cohort study. Colorectal Dis.

[14] Hindryckx P, Jairath V, Zou G, Feagan BG, Sandborn WJ, Stoker J (2019). Development and validation of a magnetic resonance index for assessing fistulas in patients with Crohn’s Disease. Gastroenterology.

[15] Fathallah N, Akaffou M, Haouari MA, Spindler L, Alam A, Barré A et al. Deep remission improves the quality of life of patients with Crohn’s disease and anoperineal fistula treated with darvadstrocel: results of a French pilot study. Tech Coloproctol. 2023. 10.1007/s10151-023-02765-7 36811811

[16] Kim J, Beek LGMM, Kyra L, van Rijn K, Horsthuis, Jeroen AW, Tielbeek, Christianne J. Buskens5, Geert R. D’Haens, Krisztina B. Gecse2, Jaap Stoker. External Validation of the MAGNIFI-CD Index in Patients with Complex Perianal Fistulising Crohn’s Disease Submitted. 10.1007/s00330-024-11029-3 PMC11836172 39212672

[17] Losco A, Viganò C, Conte D, Cesana BM, Basilisco G (2009). Assessing the activity of perianal Crohn’s disease: comparison of clinical indices and computer-assisted anal ultrasound. Inflamm Bowel Dis.

[18] Greer MC, Taylor SA (2022). Perianal Imaging in Crohn Disease: current Status with a focus on MRI, from the AJR Special Series on imaging of inflammation. AJR Am J Roentgenol.

